# 

**Published:** 1903-10

**Authors:** 


### CONTENTS.

ORIGINAL COMMUNICATIONS—

THE ETIOLOGY AND PATHOLOGICAL ANATOMY OF ANEURYSM. By W. Lowndes Peple, M.D., Richmond, Va. .... 433

CONCERNING THE SYMPTOMATOLOGY OF THORACIC ANEURYSMS. By Truman A. Parker, M.D., Richmond, Va. .... 436

GELATINE TREATMENT IN ANEURYSM. By Ennion C. Williams, M. D., Richmond, Va. .... 440

DOUBLE MAMMARY ABSCESS, WITH REPORT OF CASE. By O. L. Holmes, M.D., Stewart, Ga. .... 445

EDITORIAL NOTES AND COMMENTS—

CHARITY .... 447

MEDICAL SOCIETY TRANSACTIONS IN JOURNAL FORM .... 448

A HALLOWE'EN APPARITION .... 449

THE STATE BOARD OF HEALTH .... 450

NEWS AND NOTES .... 451

CONTENTS CONTINUED .... X

